# Human fallopian tube proteome shows high coverage of mesenchymal stem cells associated proteins

**DOI:** 10.1042/BSR20150220

**Published:** 2016-02-19

**Authors:** Chenyuan Wang, Yang Liu, Cheng Chang, Songfeng Wu, Jie Gao, Yang Zhang, Yingjie Chen, Fan Zhong, Gaopi Deng

**Affiliations:** * Gynecology Department, The First Clinical Medical School, Guangzhou University of Chinese Medicine, 12 Jichang Blvd, Guangzhou 510405, China; † Department of Systems Biology for Medicine, Shanghai Medical College, and Institutes of Biomedical Sciences, Fudan University, 138 Yixueyuan Road, Shanghai 200032, China; ‡ State Key Laboratory of Proteomics, Beijing Proteome Research Center, Beijing Institute of Radiation Medicine, Beijing 102206, China

**Keywords:** human fallopian tube, mesenchymal stem cell, oviduct, proteome

## Abstract

The object of this research was to report a draft proteome of human fallopian tube (hFT) comprises 5416 identified proteins, which could be considered as a physiological reference to complement Human Proteome Draft. The proteomic raw data and metadata were stored in an integrated proteome resources centre iProX (IPX00034300). This hFT proteome contains many hFT markers newly identified by mass spectrum. This hFT proteome comprises 660 high-, 3605 medium- and 1181 low-abundant proteins. Ribosome, cytoskeleton, vesicle and protein folding associated proteins showed obvious tendency to be higher abundance in hFT. The extraordinary high coverage of mesenchymal stem cells (MSCs)-associated proteins were identified in this hFT proteome, which highly supported that hFT should contain a plenty of MSCs.

## Abstract (precis)

We report the largest scale MS based proteome of human fallopian tube (hFT). Ribosome, cytoskeleton, and vesicle associated proteins showed high abundance in hFT. Extraordinary high coverage of MSCs associated proteins in the hFT proteome.

## INTRODUCTION

Human fallopian tube (hFT), also named oviduct, is divided into four layers: serosa, subserosa, lamina propria and the innermost mucosa in a cross section. Serosa is based on visceral peritoneum. Subserosa includes lymphatics, blood vessels, smooth muscle and loose adventitious tissue, which endows the function of peristaltic action to hFT. Lamina propria is a vascular connective tissue [1]. Two major types of cells present in the simple columnar epithelium of mucosa, secretory cells (60%) and ciliated cells (25%), in addition to <10% narrow peg cells [2]. The number of ciliated cells increases under oestrogenic effect, and peg cells secrete more tubular fluid by oestrogenic effect as well. Oviductal fluid or secreted proteins also play important roles in fertilization and early embryo development [3]. The fluid can offer nutrition to oocytes, spermatozoa and even zygotes, and help them to move smoothly in tubal. Therefore, hFT has the function of selecting spermatozoa and is vital for fertilization [4]. Protein identifications of secreted oviductal fluid have been carried out in human and many other species [5].

From the perspective of pathology, tubal ectopic pregnancy is the most focused hFT lesion, which has been explored on protein level [6]. Although a few hFT related proteome-wide researches have been carried out for pathological research purposes [7–9], there is no intact proteomic profiling of physiological hFT as reference proteome even in the Human Proteome Draft collections [10,11]. To avoid ethical issues for obtaining hFT tissue from women with healthy genital system, normal hFT can be collected from hysterectomy samples with myoma of uterus. In the present study, we have profiled proteome of hFT as a physiological reference to complement Human Proteome Draft.

## MATERIALS AND METHODS

### Samples

Tissue specimens of ampullary of hFTs were obtained from 12 patients, who were diagnosed multiple uterine myomas (ages 41–51) but with no fertility, or panhysterectomy requirements and were willing to receive salpingectomy by surgical procedure in the first affiliated hospital of Guangzhou University of Chinese Medicine. All patients were asked for consent and required to sign consents of sample collection and subsequent analysis before any procedures. They were all in follicular phase when the operation was performed. The pathological tests were performed to make sure the samples were clear of any pathology changes. Then the specimens sized 5 mm^2^ were rinsed by normal saline in 4°C, and saved in liquid nitrogen canister within 30 min after the surgery. The project was approved by the local ethics committee in the first affiliated hospital of Guangzhou University of Chinese Medicine.

### Extraction of proteins

To extract protein, we homogenized 30–50 mg tissue in 800 μl of lysis buffer that contained 7 M urea, 2 M thiourea, 50 mM DTT and protease inhibitor cocktail using tissue grinder for 2 min per sample. Then samples were centrifuged at 16000 ***g*** for 15 min at 4°C, and the supernatant liquid from each sample was obtained and stored at -80°C for further study. The protein concentration was measured by Bradford assay (Beyotime). Two milligrams of protein lysis per group were collected and separated by adding 3-fold volume cold acetone for 12–16 h with the equal protein content from each of the 12 samples. After acetone precipitation, each group of protein sample was resuspended by UA (8 M urea, 0.1 M Tris/HCl, pH 8.5), which provided a high urea concentration environment to promote protein lysis during extraction. The incubation of 400 μg of protein per group was performed for 1 h at 37°C after adding 3 μl 1 M DTT in UA, and was continued for another 1 h at room temperature in the dark after adding 15 μl 1 M IAA (for alkylation) in UA. After adding 10 kDa MWCO Vivospin (Sartorius, VS0201) ultrafiltrated, the samples were resuspended by 100 μl 1 M urea, and followed by treatment with trypsin (2–4 μg trypsin/100 μg total protein, Promega, V511C) at 37°C for 16 h. Then, another 1 μg trypsin was added in the proteins and digested at 37°C for another 6 h. This protocol is modified based on Filter Aided Sample Preparation (FASP) method [12].

### Mass spectrum based proteome profiling

The peptides were fractionated on a waters UPLC using a C18 column (Waters, BEH C18 1.7 μm, 2.1 mm X 50 mm). Peptides were eluted at a flow rate of 600 μl/min with a linear gradient of 5–35% solvent B (acetonitrile) over 10 min, the solvent A was 20 mM ammonium formate with pH adjusted to 10. The absorbance at 214 nm was monitored, and a total of 20 fractions were collected. The fraction was separated by Nano-HPLC (Eksigent Technologies) on the secondary RP analytical column (Eksigent, C18, 3 μm, 150 mm X 75 μm). Peptides were subsequently eluted using the following gradient conditions with phase B (98% ACN with 0.1% formic acid) from 5 to 45% B (5–100 min) and the total flow rate was maintained at 300 nl/min. Electrospray voltage of 2.5 kV compared with the inlet of the mass spectrometer was used.

Triple TOF 4600 mass spectrometer was operated in information-dependent data acquisition mode to switch automatically between MS and MS/MS acquisition. MS spectra were acquired across the mass range of *m*/*z* 350–1250 using 250 ms accumulation time per spectrum. Tandem mass spectral scanned from *m*/*z* 100 to 1250 in high sensitivity mode with rolling collision energy. The 25 most intense precursors were selected for fragmentation per cycle with dynamic exclusion time of 25 s. Tandem mass spectra were extracted and charge state deconvoluted by MS Data Converter from AB SCIEX. All of the MS proteomics data have also been deposited to the iProX (http://www.iprox.org/index) with the identifier IPX00034300.

Mascot (Matrix Science, London, UK; version 2.3.02) was only used to interpret samples. Mascot was set up to search the human Swiss-Prot (release 2014-09-23, 20193 entries) assuming the digestion enzyme trypsin. Mascot was searched with a fragment ion mass tolerance of 0.1 Da and a parent ion tolerance of 25 ppm. Scaffold (version 4.3.2, Proteome Software) was used to validate MS/MS based peptide and protein identifications. Peptide identifications were accepted while *FDR* less than 1.0% by the Scaffold Local *FDR* algorithm. For label-free quantification, we firstly used the same algorithm in SILVER for peptide extracted ion current (XIC) construction [13], then took the peptide XIC area as the peptide intensity. The *LFQ* value was defined as the sum of its unique peptide intensities. Finally, we carried median normalization within each replicate, and used arithmetic mean of median normalized *LFQ* values from the two replicates as the final quantification.

### DAVID enrichment analysis

The analysis was carried out using the web-accessible DAVID (database for annotation, visualization and integrated discovery) tool [14]. We upload all 5416 identified, 630 high-abundant and 1181 low-abundant proteins by UNIPROT_ACCESSION identifiers. The corresponding group-splited UniProt AC numbers were a bit higher that 5587, 643 and 1249 respectively. Default medium classification stringency was applied and the whole human genome was set as background. Enriched clusters from 640 high- and 1181 low-abundant proteins respectively were assessed by Functional Annotation Clustering in eight categories individually: functional categories, gene ontology, general annotations, literature, pathways, protein domains, protein interactions and tissue expression. We only chose five subcategories GOTERM_BP_FAT, GOTERM_MF_FAT, GOTERM_CC_FAT, PANTHER_BP_ALL and PANTHER_MF_ALL under the Gene Ontology category. For the whole identified protein list contains more than 3000 identifiers, we used Functional Annotation Chart (Thresholds: count >2, *EASE* score <0.1) to assess the enriched items in the tissue expression category.

### Pathway mapping

Ingenuity Pathway Analysis (IPA; QIAGEN, Redwood City, http://www.qiagen.com/ingenuity) was used to map all the identified hFT proteins to canonical pathways. We assigned high-, medium- and low-abundant proteins value +1, 0 and −1 respectively. These proteins would show coordinating and clustering tendencies in pathway context.

### Referenced identification datasets

We extracted 30821 identified peptides from the 11 normal hFT in Rungruang data (pr100451f_si_001–hFT.xls) [8]. There were 6453 non-redundant peptides, of which 6058 (93.9%) can be found as trypsin digested peptides in Swiss-Prot database (release 2014-09-23). The protein inference by parsimony rule gets 2580 non-redundant protein groups (Supplementary Table S2) from the 6058 peptides. Among them, 739 protein groups were identified by at least two unique peptides.

We extracted 12185 detected Ensembl genes in hFT from the Human Protein Atlas (HPA) [15]: items with annotations ‘Tissue’ of ‘fallopian tube’, ‘Expression type’ of ‘high’, ‘medium’ or ‘low’. Only 3601 of them are simultaneously with the annotation ‘Reliability’ of ‘Supportive’ rather than ‘Uncertain’. We used Hyperlink Management System to convert Ensembl gene IDs to UniProt Accession Numbers [16], and then mapped to Swiss-Prot (release 2014-09-23) items. Finally, we got 11591 Swiss-Prot items and 3624 of which are with ‘Reliability’ of ‘Supportive’.

## RESULTS

### Status of hFT proteome

**Figure 1:**
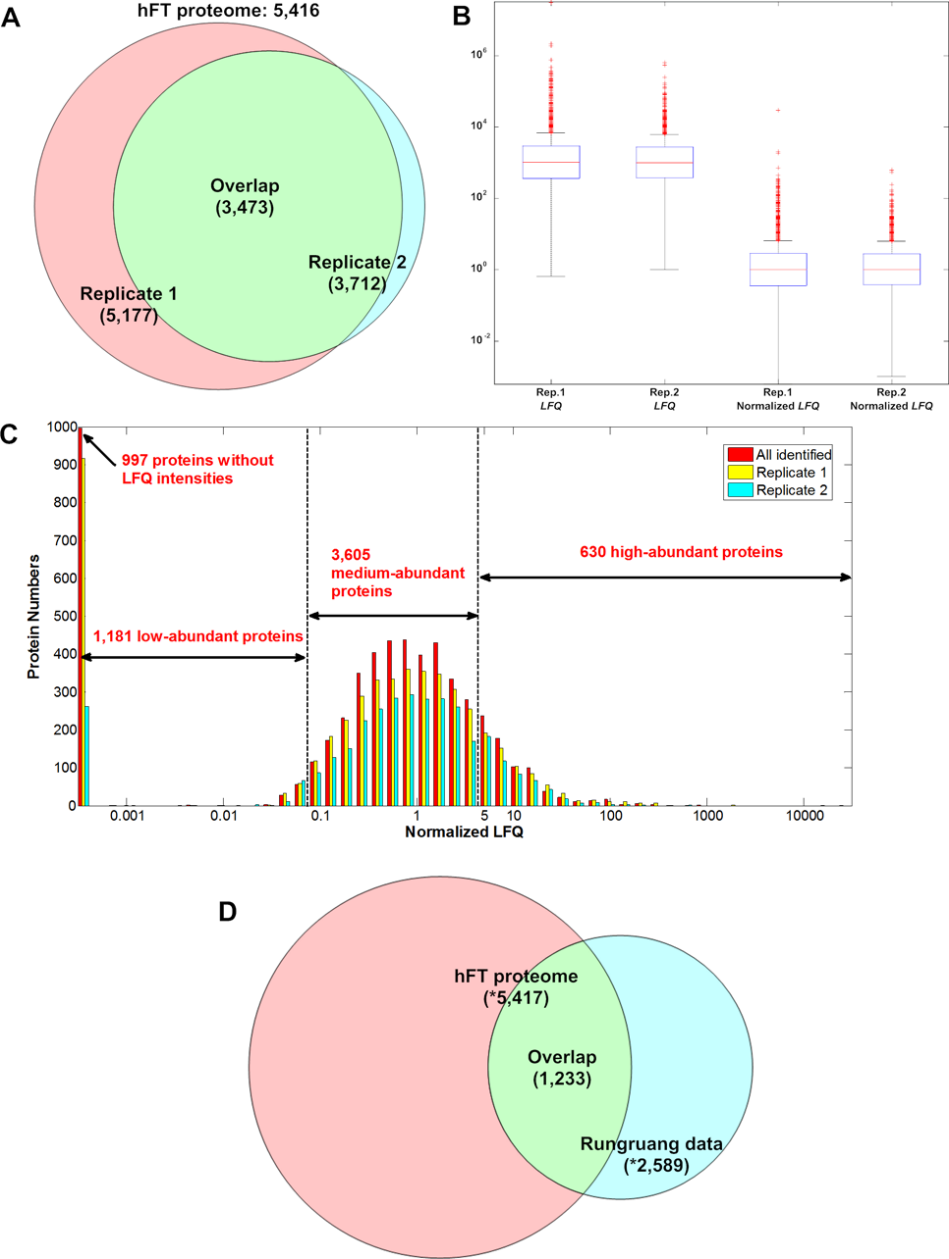
Status of hFT proteome and its overlap of Rungruang data (**A**) Two replicates were with 5177 and 3712 identified proteins respectively, totally sum up to 5416, and with 3473 overlapped. (**B**) Boxplots of original and median normalized *LFQ* values of the two replicates showed no significant difference. The median of rep. 1 and 2 were 1045 and 989 respectively. (**C**) Abundance distributions of the high-, medium- and low-abundant proteins of the whole hFT proteome and its two replicates. (**D**) The overlap of our hFT proteome and Rungruang data is merely 1233 proteins, showed high complementary of the two datasets. *The number of identified proteins was slightly more than individual statistics in both datasets, due to split of a protein group (count 1) into its members (count >1) that all were identified in the other dataset.

Replicate 1 (rep. 1) and replicate 2 (rep. 2) were with 5177 and 3712 identified proteins respectively, and were totally summed up to 5416 with 3473 overlapped (Figure 1A, see Supplementary Table S1). The most enriched tissues were caudate nucleus (*Benjamini*=3.6 × 10^−257^), epithelium (7.4 × 10^−146^), liver (2.9 × 10^−138^), platelet (4.2 × 10^−122^) and stem cell (6.8 × 10^−118^); the most overlapped tissues/cell types were adipocyte (3704 proteins), CD71+ early erythroid (3674), placenta (3568), CD14+ monocytes (3512) and CD19+ B cells (3235).

3906 and 3040 proteins of rep. 1 and rep. 2 were with valid label-free *LFQ* intensities respectively, which were summed up to 4419 proteins. We used median normalization within each replicate (Figure 1B), and further adopted arithmetic mean of the two replicates as representation of abundance. 997 proteins were identified but without valid *LFQ* intensity due to low spectrum counts or signal-to-noise. 630 proteins with abundance quantities >5 were classified as high-abundant; 3605 proteins with quantities 0.1–5 were medium-abundant; 184 proteins with quantities <0.1 and the 997 proteins with invalid *LFQ* intensities (totally 1181 proteins) were classified as low-abundant (Figure 1C).

The 630 high-abundant proteins were high enriched in stem cell (DAVID enrichment score 20.55), ribosomal (19.57), cytoskeleton (17.11), vesicle (11.75), protein folding (10.05) associated functions (Table 1). The 1181 low-abundant proteins showed moderate enrichment in nuclear lumen associated functions (6.54). Vesicle proteins of some endocytosis pathways including clathrin (Supplementary Figure S1A) and caveolar (Supplementary Figure S1B) mediated endocytosis signalling showed obvious tendency to be higher abundance. Many high-abundant proteins were also enriched in actin cytoskeleton signalling pathway (Supplementary Figure S1C). These pathway-centric phenomena were consistent to the aforementioned DAVID enrichment results.

**Table 1:** DAVID enrichment results (enrichment score in parentheses) of high- and low-abundant hFT proteins. *N/A: no item was with enrichment score more than 4 (*P*-value was around smaller than 10^−7^).

	High-abundant proteins	Low-abundant proteins
Functional categories	Transit peptide, mitochondrion (9.11)	Alternative splicing (5.28)
	Isopeptide bond (7.63)	Nucleotide-binding (5.16)
	Repeat: Spectrin (6.54)	Mitochondrion, transit peptide (4.19)
	ATP-binding (6.14)	
	Intermediate filament (5.92)	
	Repeat: Annexin (5.14)	
	Domain: Laminin EGF-like (4.29)	
Gene ontology	Cytoskeleton (17.11, 11.58)	Nuclear lumen, nucleolus (6.54)
	Pigment granule, melanosome, vesicle (11.75)	Nucleotide binding (4.61)
	Protein folding, chaperone (10.05)	Acetyltransferase activity (4.04)
	Cytoskeleton organization (7.72)	
	Mitochondrion (5.91)	
General annotations	Annexin repeats (5.50)	Histone (4.54)
Literature	Aminoacyl-tRNA synthetases (4.93)	Histone (4.17)
	ER, melanosomal (4.13)	
	Laminin-10/11 (4.11)	
Pathways	Integrin, focal adhesion, ECM-receptor (5.43)	*N/A
Protein domains	Calponin homology, Spectrin repeat (7.38)	Histone H2A (4.36)
	Intermediate filament (5.46)	
	Annexin (4.40)	
Protein interactions	Ribosomal proteins (19.57)	N/A
Tissue expression	Stem cell (20.55)	Mammary gland, breast carcinoma cell line (6.32)
	Cartilage (8.72)	Colon (5.92)
	Brain (8.43)	Monocyte (4.57)

In addition to our work, the second largest mass spectrum based hFT proteome was from the work of Rungruang et al. [8]. We obtained 2580 proteins by matching their identified peptides to Swiss-Prot release 2014-09-23 (Supplementary Table S2). The overlap of our and Rungruang data was merely 1233 proteins (Figure 1D), which showed high complementary of the two datasets. The hFT marker proteins analysis below showed our hFT proteome caught up some markers uniquely.

### Marker proteins in hFT

A plenty of hFT markers had been identified in our dataset including mucins (MUC1) [17], oviduct specific glycoprotein 1 (OVGP1) [18], beta-tubulin IV (TUBB4B) [19], progesterone receptor (PGR) [20], membrane-associated progesterone receptor component 1 (PGRMC1) and 2 (PGRMC2) [20], oestrogen-related receptor gamma (ESRRG), inhibin alpha chain (INHA) [21], beta A chain (INHBA) [21], endothelial nitric oxide synthase (NOS3) [22], nitric oxide synthase-interacting protein (NOSIP) [22] and epidermal growth factor receptor (EGFR) [23]. Seven markers, PGRMC1, PGRMC2, ESRRG, INHA, INHBA, NOS3 and NOSIP, were newly identified by our dataset compared with Rungruang data. However, there were still some hFT markers missed in our dataset such as PAX8 [24], activins [25], adrenomedullin (ADM) [26], prokineticins (PROK1, PROK2) and their receptors (PROKR1, PROKR2) [27].

### Mesenchymal stem cells (MSCs) associated proteins

One of the most noticeable phenomena of hFT proteome was that stem cell associated proteins were enriched significantly (*Benjamini*=6.8 × 10^−118^). The enrichment was even more prominent in the 630 high-abundant proteins, which scored 20.55 in ‘stem cell’ and was far greater than the other enriched tissue/organ/cell expressions (Table 1). The results indicated that stem cell associated proteins were not just widely but also highly expressed in hFT.

Zatz and colleagues reported hFT was a great rich source of mesenchymal stem cells (MSCs), and identified MSCs protein markers CD13, CD29, CD44, CD73 (SH3, SH4), CD90, CD105 (SH2) and HLA-ABC [28]. They also used hFT sourced mesenchymal stromal cells to treat osteoporosis [29]. The hFT proteome in this work has identified most of the MSCs protein markers, only except for CD13, CD19, CD79A, CD116, CD117, CD133, CD349, SOX11 and TM4SF1 (Table 2) [30]. The result highly supported that hFT should contain a plenty of MSCs.

**Table 2:** Identification status of the 35 MSCs associated proteins in hFT proteome. *Unidentified: unidentified in this hFT proteome. †N/A: identified but without valid *LFQ* intensity assignment.

Markers	Gene Name	Uniprot AC	Abundance
CD10	*MME*	P08473	2.983
CD13	*ANPEP*	P15144	*Unidentified
CD14	*CD14*	P08571	9.413
CD19	*CD19*	P15391	Unidentified
CD29	*ITGB1*	P05556	6.659
CD31	*PECAM1*	P16284	1.661
CD34	*CD34*	P28906	†N/A
CD38	*CD38*	P28907	0.303
CD44	*CD44*	P16070	1.189
CD45	*PTPRC*	P08575	1.954
CD49f	*ITGA6*	P23229	5.211
CD56	*NCAM1*	P13591	2.367
CD73	*NT5E*	P21589	4.281
CD79A	*CD79A*	P11912	Unidentified
CD90	*THY1*	P04216	0.412
CD104	*ITGB4*	P16144	1.956
CD105	*ENG*	P17813	2.490
CD116	*CSF2RA*	P15509	Unidentified
CD117	*KIT*	P10721	Unidentified
CD133	*PROM1*	O43490	Unidentified
CD146	*MCAM*	P43121	2.414
CD200	*CD200*	P41217	N/A
CD271	*NGFR*	P08138	N/A
CD349	*FZD9*	O00144	Unidentified
HLA-A	*HLA-A*	P04439; Q09160; P13746	0.433
HLA-B	*HLA-B*	Q29836; P30475;	N/A
		Q95365; P18463	
HLA-C	*HLA-C*	P30499	0.115
HLA-DRA	*HLA-DRA*	P01903	4.510
HLA-DRB1	*HLA-DRB1*	P01911	N/A
		Q9TQE0	1.321
MSCA-1	*ALPL*	P05186	0.119
NG2	*CSPG4*	Q6UVK1	3.981
PODXL	*PODXL*	O00592	1.712
SOX11	*SOX11*	P35716	Unidentified
SUSD2	*SUSD2*	Q9UGT4	0.295
TM4SF1	*TM4SF1*	P30408	Unidentified

## DISCUSSION

In the present study, we have profiled a hFT proteome from hysterectomy samples, providing a reference for future investigations of hFT. The expression status of some identified proteins are suggestive of their functions and involvement in pathophysiology of hFT. Vesicle proteins of clathrin and caveolar mediated endocytosis signalling pathways showed high abundance tendency in our results. It may associate with NOS3 storage mecha-nism in hFT. NOS3, which plays an important role of nitric oxide (NO) release in human endothelial cell. NOS3 is usually stored in caveolin-1 (CAV1) and further activated by phosphorylation [31]. Also, NOS3 in Ca^2+^-dependent activation is regulated by caveolae internalization [32]. Many high-abundant proteins in our dataset were also enriched in actin cytoskeleton signalling pathway. Cytoskeleton is related to contraction of smooth muscle that depends on dense bodies in the cytoplasm and adherens junctions in cytoplasmic face of the sarcolemma to interact with muscle cells [33]. The motility function of hFT relies on its smooth muscle, so cytoskeleton signal is crucial in the physiological function of fertility as well.

Mucins are large glycoproteins that coat the surface of cells in respiratory, digestive, and urogenital tracts. Gipson et al. has systematically compared the expression status of mucin members in human female reproductive tract epithelia, and found that MUC1 was the only mucin express in hFT and as a marker for epithelial secretory cells. Our data have repeated this phenomenon: MUC1 was the only mucin identified in hFT. OVGP1 is an iconic protein in oviductal secretory cells [18], and TUBB4B is an essential component of cilia in the oviduct [19]. Our data have identified OVGP1 and TUBB4B in both two replicates, caught sample features from both secretory and ciliated oviductal epithelium. PGR, PGRMC1 and PGRMC2 were all identified. ESRRG was the only identified member of oestrogen receptors (ERs) in our data. The fluctuating levels of sex steroids (progesterone, oestrogen and androgen) affect female reproductive system during the normal menstrual cycle. Down-regulation of PGR plays an important role of successful embryo-tubal transport or subsequent implantation in the uterus, by inhibiting the ciliary and tubal smooth muscle activity. ERs in the physiology of hFT, may reduce expression of protein by whether enhance degradation or reduce translation. Expression patterns of ERs, PGR and androgen receptor (AR) have yet to be comprehensively studied in the normal hFT or in the context of tubal pathologies [20]. Other hFT marker proteins such as INHA, INHBA, NOS3 and NOSIP have been identified in our hFT proteome but was missed in Rungruang data. Inhibins are members of the TGFβ superfamily, which consist of an alpha subunit (inhibin alpha). Inhibin alpha is a kind of heterodimeric peptide. It is predominately secreted by the corpus luteum. Inhibins proteins displaced its own receptors to counteract activins [21].

Nitric oxide is produced by the reaction of L-arginine, when nitric oxide synthase (NOS) catalysed reaction. NOS contains three isoforms: endothelial (eNOS, NOS3), neural (nNOS, NOS1) and inducible nitric oxide synthase (iNOS, NOS2) [22]. NO can relax tubal smooth muscle as it is expressed in hFT. iNOS protein were verified in hFT [34], and raises its level may increase frequency of ciliary beat or altered the contractility of fallopian tube smooth muscle. EGFR has been identified in both our hFT proteome and Rungruang data. EGFR is the cell-surface receptor, belonging to the members of the epidermal growth factor (EGF) family of extracellular protein ligands. EGFR proteins are shown as modulate phenotypes: adhesion, cell migration and proliferation [35]. EGFR was localized to the epithelium in the ampullary portion of fallopian and showed that specific staining by immunohistochemical studies [23]. Although, the major function of EGF in hFT epithelial cells are still unknown, and aberrant EGFR signalling may lead to human tubal ectopic pregnancy.

There were still some missed proteins that are believed to express in hFT: PAX8, a marker of fallopian tube secretory cell [24], but missed in our identified list and also Rungruang data. In the fallopian tubal epithelium, PAX8 is a marker of the secretory cell lineage, not the ciliated cell population [36]. We assume that the lack of PAX8 identification might be due to the relative small fraction of secretory cells exist in hFT samples. Activins, ADM, prokineticins (PROK1, PROK2) and their receptors (PROKR1, PROKR2) were all missed in our and Rungruang data. Activins and their receptors have been identified for their activating effect in hFT by using immunohistochemistry [25]. Activins can also increase NOS productions to add the motility of hFT [37]. AMD belongs to peptide hormone in the calcitonin or calcitonin gene-related peptide amylin family, and can promote angiogenesis. Expression of ADM in fallopian tube was verified in the study about the pathogenesis of tubal ectopic pregnancy [26]. Prokineticins are the cognate ligands of two homologous G protein-coupled receptors, and can induce angiogenesis and contractions of smooth muscle [38]. Prokineticins and their receptors are localized to the smooth muscle of hFT [27]. PROK1 can up-regulate cyclooxygenase-2 [39], and further increases the contractility of smooth muscle of hFT [40]. PROK2 and PROKR1 mRNA have been reported more highly expressed in hFT from the P-dominant, midluteal phase compared with the follicular phase [27]. All the samples in our experiment were from follicular phase, so the missed identification of prokineticins and their receptors in our dataset were possibly due to their expression dependence of the hFT hormonal status. We can also find immunohistochemistry references from the HPA [15]: activin A receptor type IB (ACVR1B), type IC (ACVR1C), type II-like 1 (ACVRL1), PROK1 and PROK2 were not detected; PAX8 and ADM were of low abundance; activin A receptor type I (ACVR1), type IIA (ACVR2A), type IIB (ACVR2B), PROKR1 and PROKR2 were of medium abundance in hFT. In consideration of immunohistochemistry detection is in principle more sensitive than mass spectrums, the lack of these hFT markers in our data was likely due to their low abundances. The HPA detected 12185 Ensembl genes in hFT by immunohistochemistry arrays, which can be mapped to 11591 non-redundant Swiss-Prot (release 2014-09-23) items. These results suggest that although our hFT proteome and Rungruang data have showed high complementary, there is still a considerable space for us to achieve a complete hFT proteome. Relying on the current techniques, further deeply covered proteome profiling of multiple fallopian tube tissues from individuals will allow us to approach a possible saturated mass spectrum detected proteome of hFT.

MSCs are a subset of stromal cells in adult connective tissues but always at low presentation frequencies [41], and have the capacity to differentiate into cells of connective tissue lineages [42]. hFT contains a large amount of mesenchymal connective tissues, which properly offers the suitable niches for the residence of MSCs [43]. Although it is not the first reveal of wealthy MSCs resident in hFT [28], our data offer a sufficient support in proteome level.

## Abbreviations

ADM: adrenomedullin
DAVID: database for annotation, visualization and integrated discovery
ER: oestrogen receptors
hFT: human fallopian tube
HPA: human protein atlas
iNOS: inducible nitric oxide synthase
INHA: inhibin alpha chain
INHBA: inhibin beta A chain
IPA: ingenuity pathway analysis
MSC: mesenchymal stem cell
NOS: nitric oxide synthase
NOSIP: nitric oxide synthase-interacting protein
XIC: extracted ion current
